# Measuring the density structure of an accretion hot spot

**DOI:** 10.1038/s41586-021-03751-5

**Published:** 2021-09-01

**Authors:** C. C. Espaillat, C. E. Robinson, M. M. Romanova, T. Thanathibodee, J. Wendeborn, N. Calvet, M. Reynolds, J. Muzerolle

**Affiliations:** 1 Institute for Astrophysical Research, Department of Astronomy, Boston University, Boston, MA USA; 2 Department of Physics and Astronomy, Amherst College, Amherst, MA USA; 3 Department of Astronomy, Cornell University, Ithaca, NY USA; 4 Department of Astronomy, University of Michigan, Ann Arbor, MI USA; 5 Space Telescope Science Institute, Baltimore, MD USA

**Keywords:** Astronomy and planetary science, Astrophysical disks

## Abstract

Magnetospheric accretion models predict that matter from protoplanetary disks accretes onto stars via funnel flows, which follow stellar magnetic field lines and shock on the stellar surfaces^1–3^, leaving hot spots with density gradients^4–6^. Previous work has provided observational evidence of varying density in hot spots^7^, but these observations were not sensitive to the radial density distribution. Attempts have been made to measure this distribution using X-ray observations^8–10^; however, X-ray emission traces only a fraction of the hot spot^11,12^ and also coronal emission^13,14^. Here we report periodic ultraviolet and optical light curves of the accreting star GM Aurigae, which have a time lag of about one day between their peaks. The periodicity arises because the source of the ultraviolet and optical emission moves into and out of view as it rotates along with the star. The time lag indicates a difference in the spatial distribution of ultraviolet and optical brightness over the stellar surface. Within the framework of a magnetospheric accretion model, this finding indicates the presence of a radial density gradient in a hot spot on the stellar surface, because regions of the hot spot with different densities have different temperatures and therefore emit radiation at different wavelengths.

## Abstract (web summary)

An observed one-day difference between the peaks of emission of ultraviolet and optical light from the hot spot on GM Aurigae indicates that the hot spot has a radial density gradient.

## Main

We conducted a coordinated multiepoch multiwavelength observing campaign of the accreting T Tauri star GM Aur, a roughly 2-Myr-old solar analogue that is surrounded by a disk with a large cavity with a radius of about 20 au^15–17^. Gas is flowing inside the cavity and eventually reaches the star, as evidenced by moderate accretion rates^18^; however, most of the solid material in the cavity has grown to millimetre and larger sizes, as indicated by the lack of substantial infrared emission^15^ that would be present if a substantial amount of smaller dust grains remained. Because giant planets must be present for large cavities (tens of astronomical units in size^19,20^) to open in protoplanetary disks, GM Aur is an ideal candidate to study a protoplanetary disk with the properties required to form planets.

**Fig. 1:**
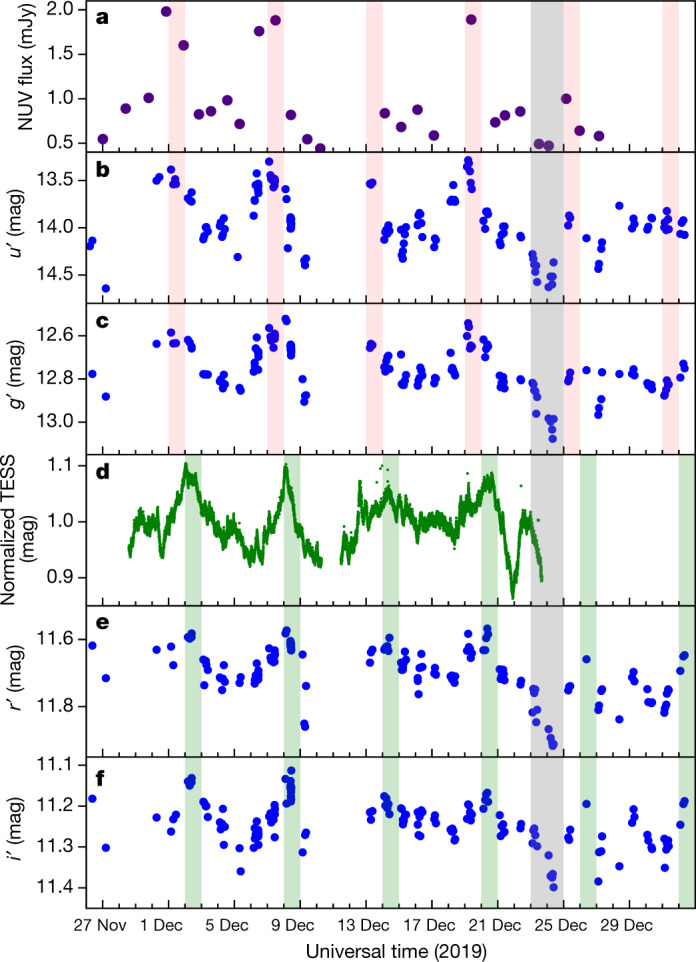
Multiwavelength light curves of GM Aur. **a**–**f**, Swift NUV fluxes (**a**; Extended Data Table 1), LCOGT *u*′*g*′ flux calibrated magnitudes (**b**, **c**), TESS normalized magnitudes (**d**) and LCOGT *r*′*i*′ flux calibrated magnitudes (**e**, **f**). The TESS bandpass covers the bandpass of the *r*′ and *i*′ data. Uncertainties are smaller than the symbols. Red and green shaded boxes highlight peaks in the UV and optical light curves, respectively, and are separated by the rotation period of the star. Dark grey shaded boxes highlight a contemporaneous dip in all the light curves.

**Extended Data Fig. 1:**
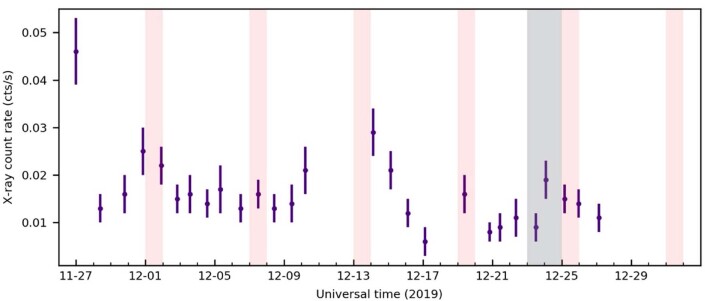
X-ray count rates of GM Aur. Data were observed by Swift between 2019 November 26 and 2020 January 1, and are listed in Extended Data Table 1. To facilitate comparison, we use the same time range and colour bars as in Fig. 1 for the Swift NUV data. Error bars correspond to uncertainties, which are listed in Extended Data Table 1.

Here we present results from our multiwavelength variability study, including Neil Gehrels Swift Observatory (Swift) X-ray and near-ultraviolet (NUV) fluxes, Hubble Space Telescope (HST) NUV spectra, Las Cumbres Observatory Global Telescope (LCOGT) *u*′*g*′*r*′*i*′ photometry, Transiting Exoplanet Survey Satellite (TESS) photometry and CHIRON Hα spectra taken contemporaneously over roughly 35 days. We find no significant variation in the X-ray emission (Extended Data Fig. 1) and therefore rule it out as being responsible for any of the changes seen and do not discuss it further. Daily changes appear in the Swift NUV, LCOGT *u*′*g*′*r*′*i*′ and TESS light curves, with a period of about 6 days (Methods), consistent with the 6.1-day rotation period of the star^21^ and evidence of rotational modulation (Fig. 1). The NUV, *u*′, and *g*′ fluxes (Fig. 1, red shaded boxes) peak about a day before (Methods) the TESS, *r*′ and *i*′ fluxes (Fig. 1, green shaded boxes). Specifically, the UV data peaked on 2019 December 1, 7, 13 (not as well defined owing to sparse data) and 19, whereas the optical data peaked on 2019 December 2, 8, 14 (not as well defined in the TESS light curve) and 20. There is also a dip in all the light curves on December 23–25 (Fig. 1, grey shaded boxes). This is followed by the disappearance of the UV peaks in the light curves; the optical peaks remain, but appear to be lower than those before the dip in the light curves (Fig. 1). We propose that this combination of features in the light curves is due to structure in the hot spot.

The time lag between the peaks in the UV and optical light curves and the eventual disappearance of the UV peaks is the first observational evidence of a density gradient in a hot spot on a stellar surface. The UV emission traces the high-density region of the hot spot and the optical emission traces the low-density region^7^. As the star rotates, we see the different density regions of the hot spot as it goes in and out of view. When the high-density region of the hot spot disappears, the UV emission decreases substantially.

**Fig. 2:**
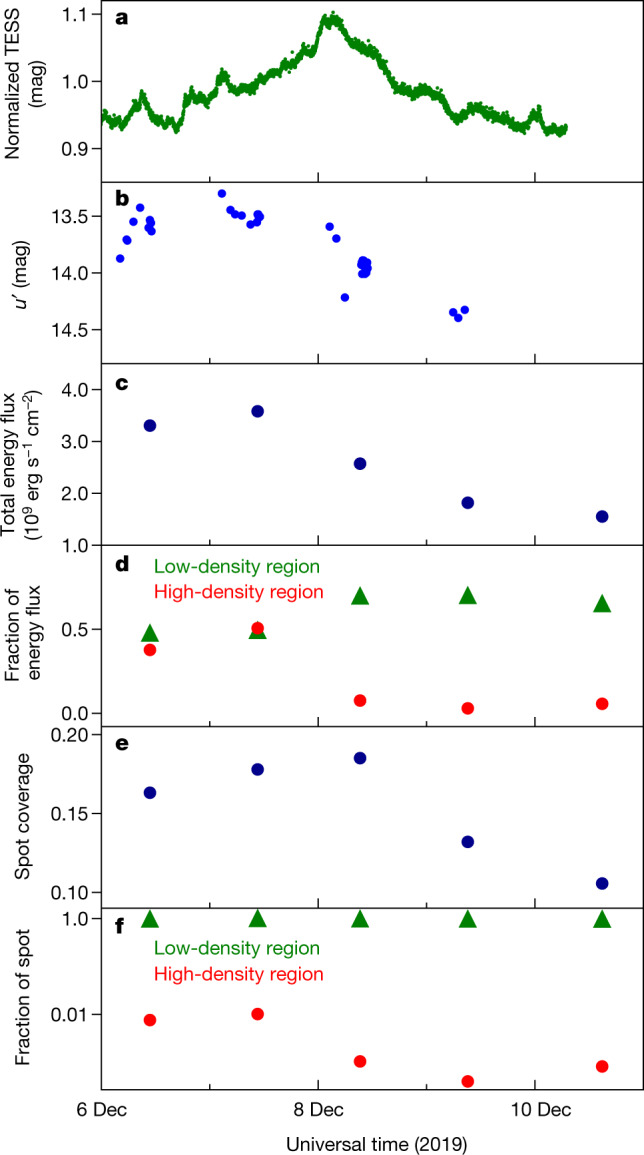
Comparison of simulated hot-spot properties to observed light curves. Best-fitting accretion-shock model parameters (Extended Data Table 2) are derived by fitting HST spectra (Methods). **a**, **b**, TESS optical light curve (**a**) and LCOGT *u*′ light curve (**b**) between 2019 December 6 and 10. **c**, **d**, When *u*′ peaks, the total energy flux of the accretion column (**c**) and the contribution from the high-density region (**d**) are highest. **e**, The size of the hot spot peaks along with the optical data. **f**, The low-density region dominates on all days, whereas the high-density region drops substantially after 2019 December 8.

Accretion-shock modelling confirms that the high-density region of the hot spot dominates the UV emission and that the low-density region of the hot spot dominates the optical emission (Methods). These models consist of a vertical accretion column close to the surface of the star that is constrained by the stellar magnetic field and divided into three subregions: the pre-shock zone, the post-shock zone and the heated photosphere below the shock^22^. The top of the accretion column is met by the funnel flow and the base is the hot spot on the stellar surface. There is a peak in both the observed UV emission and the model emission from the high-density region of the hot spot about 1 day before the peak in both the observed optical emission and the model emission from the low-density region (Fig. 2). Periodicity in the light curves (Methods) consistent with the stellar rotation period^21^ points to rotational modulation. This supports our contention that we are seeing a hot spot with a density gradient as it rotates along with the star.

The above is also consistent with magnetohydrodynamic simulations, which predict that there should be a density gradient in the accretion flow, column and hot spot^5,8,23^. We quantitatively compare the accretion-shock modelling results to the size of the hot spot predicted by three-dimensional magnetohydrodynamic models^5^. The low-density region of the hot spot is predicted to cover 10%–20% of the stellar surface. At high-density, this coverage can drop to less than 1%. Our accretion-shock modelling shows that the surface coverage of the hot spot decreases at larger densities, consistent with the theoretical predictions. Specifically, the coverage of the low-density region is 10%–20% and the coverage of the high-density region is 0.1% (Fig. 2, Extended Data Table 2, Methods). The sizes of these accretion hot spots are also roughly consistent with those measured from Zeeman Doppler imaging of young stars^24^.

**Fig. 3:**
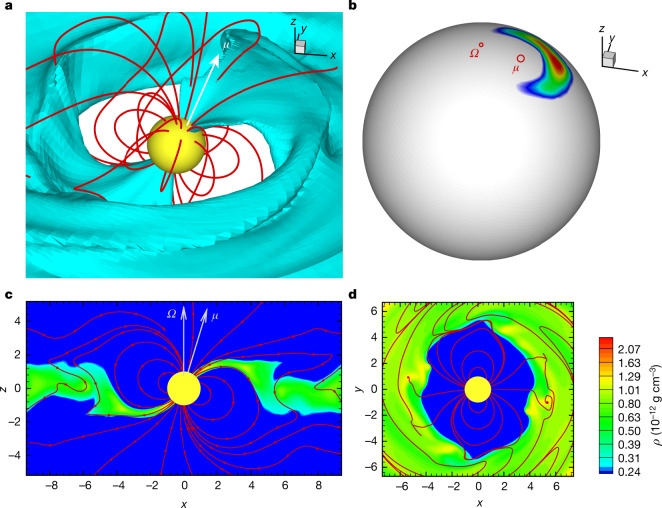
Three-dimensional magnetohydrodynamic simulations of accretion. **a**, A three-dimensional view of matter at a density level of 5.3 × 10^−13^ g cm^−3^ (blue) flowing onto the star (yellow) along selected magnetic-field lines (red) at 3.13*P*_star_ (where *P*_star_ is the stellar rotation period). Distance is measured in stellar radii (*R*_star_). Only the central region (42*R*_star_) is shown. **b**–**d**, The corresponding hot spot (**b**), along with slices in the *x*–*z* (**c**) and *x*–*y* (**d**) planes; the colour scale denotes the density. Here *μ* is the magnetic moment, *Ω* is the rotation axis and *ρ* is the density.

**Fig. 4:**
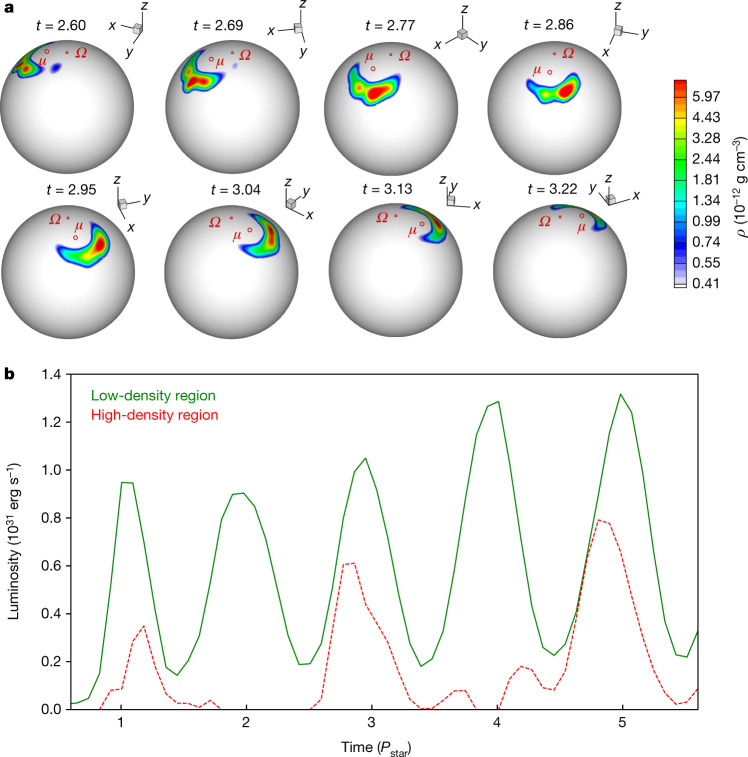
Simulations of the hot spot and light curves. **a**, The hot spot is crescent-shaped, is bent around the magnetic pole and has a density gradient. Here *μ* is the magnetic moment, *Ω* is the rotation axis, *ρ* is the density and *t* is time. **b**, The light curves generated by the less dense (solid) and densest (broken) regions of the hot spot are modulated by the stellar rotation period (*P*_star_). The high-density region of the hot spot rotates out of view, leading to a time lag between the light curves at about 3*P*_star_ and 5*P*_star_; it may also occasionally disappear (at about 2*P*_star_ and 4*P*_star_).

We also qualitatively compare the observed light curves with three-dimensional magnetohydrodynamic simulations that have the approximate properties of GM Aur (Fig. 3, Methods). Our observations point to a hot spot with a density gradient where at times the smaller high-density region of the hot spot is no longer visible (presumably because it is behind the star) while the larger low-density region is still visible. This is consistent with three-dimensional magnetohydrodynamic simulations^5,23,25^, which predict that there may be a density gradient in the hot spot and that at times parts of the hot spot may not be visible owing to stellar rotation (Fig. 4, top). The observations are also consistent with simulated light curves (Fig. 4, bottom). Like the observations, the simulated light curves are modulated mainly by the stellar rotation, and the emission from the high-density region of the spot peaks before the emission from the low-density region. Another piece of evidence of structure in the hot spot is the disappearance of the UV peaks in the light curves after December 25, while the optical peaks appear to remain (Fig. 1). The position, shape and structure of the hot spot varies with time, which particularly influences the high-density region of the hot spot, causing it to occasionally disappear (Fig. 4, Methods). Therefore, emission from the low-density region of the hot spot persists while emission from the high-density region of the hot spot may disappear, as observed here.

The time delay between the peaks in the UV and optical data rules out the possibility that the hot spot is azimuthally symmetric. However, the simulations do not find that the hot spot is systematically asymmetric. Simulations show that the spots may become asymmetric owing to complex processes at the disk–magnetosphere boundary (Fig. 4, Methods). The observed systematic behaviour may result from a more complex multipole magnetic field closer to the star, which redirects part of the funnel flow such that the spots become systematically asymmetric in the azimuthal direction^26,27^. Previous three-dimensional simulations of stars with dipole and octupole fields show that if the octupole field dominates near the star it may redirect the flow and shape the hot spot^28^.

There are dips in the Swift, LCOGT and/or TESS light curves on December 21–22 and December 23–24 that precede the disappearance of the UV peaks. The first dip on December 21–22 is narrow and seen only in the TESS data (the Swift and LCOGT observations did not overlap in time). We see the start of the second dip on December 23 in the TESS data and we trace the entire dip in the Swift and LCOGT data (Fig. 1, dark grey shaded boxes). Dust extinction may lead to dips in the light curve^29^; however, GM Aur does not have substantial dust in the inner disk^15^. The observed dips are close to the expected minimum flux due to stellar rotation. There may have also been a decrease in accretion and hence hot-spot emission around this time, leading to a corresponding decrease at UV and optical wavelengths. This is consistent with the subsequent disappearance of the UV peaks and the lower optical peaks.

## Methods

### Observations and data reduction

Our campaign of GM Aur was undertaken mostly during 2019 December. Here we present data spanning the X-ray to the optical from Swift, HST, LCOGT, TESS and CHIRON. We provide details on the data reduction below.

Swift observations were taken with the X-ray Telescope^30^ and the Ultraviolet and Optical Telescope^31^, using the UVM2 filter (2,221 Å), daily between 2019 November 27 and 2019 December 27, except on days when observations could not be scheduled. For the 27 observations, observation IDs, exposure times, start times, X-ray count rates, NUV fluxes and measurement uncertainties are listed in Extended Data Table 1. The observation IDs are as follows: 0034249045–0034249058, 0034249060–0034249063, 0034249065–0034249070 and 0034249072–0034249074. We used the High Energy Astrophysics Science Archive Research Center (HEASARC) HEASoft software (version 6.22.1) to measure count rates and NUV fluxes. X-ray emission from the stellar corona is known to be variable^32,33^. X-ray count rates (0.5–10.0 keV) of GM Aur usually varied between 0.006 counts s^−1^ and 0.029 counts s^−1^ (Extended Data Fig. 1) and are comparable to those previously seen in GM Aur^34^, pointing to an X-ray luminosity of around 3 × 10^30^ erg s^−1^ (assuming a distance of around 160 pc^35^) throughout the campaign. The count rate was highest on 2019 November 27 (0.046 counts s^−1^). None of the X-ray variability observed seems to correlate with any other data obtained during this campaign (Fig. 1, Extended Data Fig. 1) and so we do not discuss it further.

**Extended Data Fig. 2:**
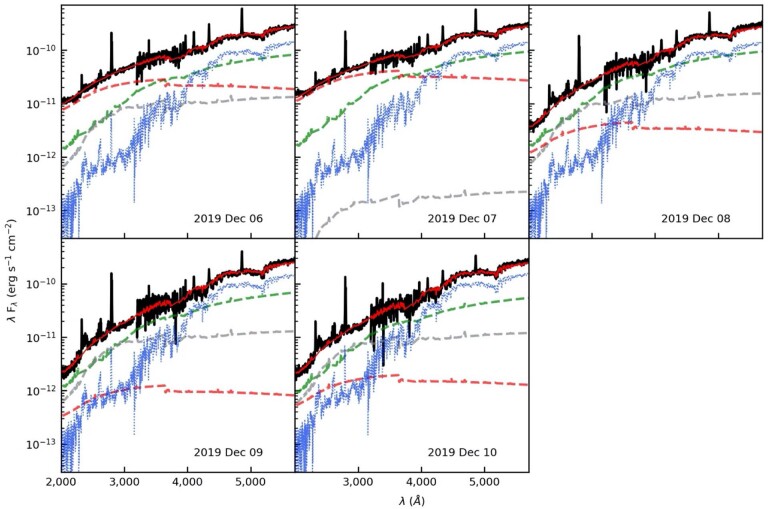
Accretion shock modelling. We show the best-fitting models (red solid line) to HST NUV–NIR spectra (black solid line). The total model consists of a non-accreting template star (blue dotted line) and three components with energy fluxes of 1 × 10^10^ erg s^−1^ cm^−2^ (green dashed line), 1 × 10^11^ erg s^−1^ cm^−2^ (grey dashed line) and 1 × 10^12^ erg s^−1^ cm^−2^ (red dashed line). Best-fitting model parameters are listed in Extended Data Table 2. *λ* and *F*_*λ*_ are the wavelength and flux density, respectively.

HST NUV–near-infrared (NIR) spectra (1,700–10,000 Å with a resolution, *R*, of 500–1,000) were taken with the Space Telescope Imaging Spectrograph (STIS) on 2019 December 6, 7, 8, 9 and 10 (Extended Data Fig. 2) in programme 16010. The spectra were obtained with the G230L, G430L and G750L gratings, with a 52″ × 2″ slit, within one orbit per visit. Data were reduced automatically by the HST pipeline; we correct fringing in the G750L data^36^. Archival G230L and G430L spectra of the non-accreting star RECX 1 (programme 11616) were used as the template for the accretion-shock modelling analysis below. Typical uncertainties on the HST spectra are 3%–15%.

TESS data were taken on 2019 November 28 to 2019 December 23 (sector 19), with a cadence of 2 min (Fig. 1). The TESS bandpass covers 6,000–10,000 Å and is centred at 7,865 Å. Data were reduced using the Science Processing Operations Center pipeline. We used Lightkurve^37^ to check the data quality and found little contamination from nearby sources. The gap in the data is due to data download during orbital perigee. Typical uncertainties in the TESS data are 0.25%.

*u*′*g*′*r*′*i*′ data were taken roughly 5–10 times on clear nights at the LCOGT^38^, with the Sinistro Imagers on the 1-m telescopes, between 2019 November 26 and 2020 January 1. The *u*′*g*′*r*′*i*′ filters have central wavelengths of 3,540 Å, 4,770 Å, 6,215 Å and 7,545 Å, and wavelength widths of 570 Å, 1,500 Å, 1,390 Å and 1,290 Å. Data in Fig. 1 were reduced using the Aperture Photometry Tool and standard aperture photometry techniques. Uncertainties in all bands are less than 0.005 mag. The flux of GM Aur was calibrated using the fluxes of background objects, which in turn were flux-calibrated using UBVRI data of background objects (converted to *u*′*g*′*r*′*i*′^39^) and the GD 64 standard field^40^. These calibration data were taken contemporaneously at the 4.3-m Lowell Discovery Telescope (LDT), using the Large Monolithic Imager, on 2019 December 2, 7, 10, 13, 18 and 21. The LDT data are used here only to flux-calibrate the LCOGT data.

**Extended Data Fig. 3:**
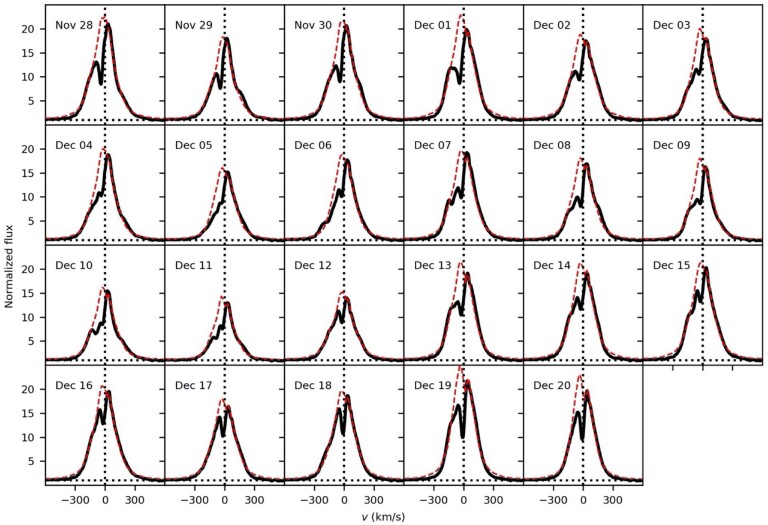
Accretion flow modelling. We show the best-fitting models (red dashed lines) to Hα line profiles (black solid lines) measured with CHIRON and plotted in velocity, *v*. Best-fitting model parameters are listed in Extended Data Table 3. There is evidence for a wind that is absorbing part of the Hα line profile on the blue side.

Medium-resolution (*R* ≈ 25,000) optical (4,082–8,906 Å) spectra were obtained with CHIRON^41^, on a 1.5-m telescope that is part of the Small and Moderate Aperture Research Telescope System (SMARTS) at Cerro Tololo Inter-American Observatory, between 2019 November 28 and December 17 (Extended Data Fig. 3). The standard CHIRON pipeline is not optimized to extract the Hα profile of young stars; we reduce the spectra here^42^.

### Timing analysis

Although the periodicity and time lag in the light curves are evident (Fig. 1), here we measure the period of the TESS and LCOGT *u*′*g*′*r*′*i*′ light curves as well as the time lag between the peak in the *u*′ light curve and those in the *g*′*r*′*i*′ and TESS light curves.

The Swift NUV data are much sparser, and we do not analyse them in as much detail.

To measure the period in the light curves, we use the Astropy Lomb–Scargle periodogram function^43–45^. We obtain a 5.8-day period for the TESS light curve; for the *u*′*g*′*r*′*i*′ light curves, we measure periods of 6.3, 6.3, 6.3 and 6.1 days. The Swift NUV flux is greater than 50% higher than surrounding days on November 30–December 1, December 7–8 and December 19 (Fig. 1), which is consistent with a roughly 6-day period. Given that the independently measured rotation period of the star is 6.1 days^21^, we attribute the roughly 6-day period measured in the light curves to stellar rotation.

For the time-lag analysis, we use the Python package Stingray^46^. When compared to the *u*′ light curve, we measure time lags of around 0, 0.75, 1.1 and 1 days for the *g*′*r*′*i*′ and TESS light curves. The Swift NUV flux is highest on the same days as the peaks seen in the *u*′ light curve (Fig. 1). We conclude that the time lag between the UV and optical data is about 1 day.

### Accretion-shock modelling

To further explore the roughly 1-day time lag in the peak between the UV and optical data seen in Fig. 1, we focus on 2019 December 6–10 (Fig. 2a, b) because HST data were taken daily during this time. HST NUV spectra are the best measure of accretion because the accretion column that channels material onto the surface of the star emits substantial energy at NUV wavelengths^1^.

The HST data were fitted using accretion-shock models^22^. These models provide information on the physical properties of the accretion column and the associated hot spot, which is the footprint of the accretion column on the stellar surface. The accretion column is characterized by an energy flux (*ρv*_s_^3^/2), which measures the density of material in the accretion column (*ρ*), assuming that the magnetospheric radius (*R*_mag_) and infall velocity (*v*_s_; here 456 km s^−1^, which depends on the stellar radius *R*_star_, stellar mass *M*_star_ and *R*_mag_) are constant. Each column has a filling factor *f*, which gives the fraction of the stellar surface covered by the column (that is, the hot spot).

GM Aur is a well studied source in the NUV, with eight previously modelled epochs^18^. In Fig. 2c–f, we plot the accretion-column model parameters (Extended Data Table 2) obtained from fitting the HST data (Extended Data Fig. 2). The fitting was done with three accretion columns^7,18^, with energy fluxes of 1 × 10^10^ erg s^−1^ cm^−2^, 1 × 10^11^ erg s^−1^ cm^−2^ and 1 × 10^12^ erg s^−1^ cm^−2^, and adopting published stellar parameters^47^. Here we refer to the 1 × 10^10^ erg s^−1^ cm^−2^ and 1 × 10^12^ erg s^−1^ cm^−2^ components as the ‘low-density’ and ‘high-density’ regions. The total energy flux (*F*_total_; Fig. 2c) is the sum of the energy fluxes of all the regions, weighted by their respective *f*. The total hot-spot coverage on the stellar surface (Fig. 2e) is the sum of the *f* values for all the regions.

The total energy flux peaks along with the UV data (Fig. 2c). When the energy flux peaks on December 7, it is dominated by emission from a high-density region, which then drops substantially (Fig. 2d). This suggests that on December 7 we see the high-density region of the hot spot and that in the following three days most of this high-density region is no longer visible. Meanwhile, the hot-spot coverage of the star peaked about 1 day later, on December 8, along with the optical data (Fig. 2e), and the hot spot is dominated by emission from a low-density region (Fig. 2f). Because the low-density region of the accretion column emits its energy at longer wavelengths^7^, it follows that the optical emission peaks when the hot spot is at its largest. The observed behaviour in the light curves may be interpreted by combining two effects: the stellar rotation and the different physical locations for the high-density and low-density regions. It then follows that the high-density region of the hot spot (whose energy appears mainly in the UV) will lead to a peak in the UV data when the high-density parts of the hot spot are visible to the observer. As the star rotates, we may be seeing a denser part of the hot spot first, which then rotates out of view.

### Accretion-flow modelling

To facilitate comparison to the three-dimensional (3D) magnetohydrodynamic (MHD) simulations, we need to estimate the magnetospheric radius of GM Aur. Magnetospheric accretion-flow modelling^48–50^ of the Hα line provides properties of the accretion flow. The model assumes that the magnetic, stellar-rotation and disk-rotation axes are aligned. The material flows onto the star along an axisymmetric accretion flow that arises from the co-rotating gas disk. The geometry of the flow is described by a dipolar magnetic field and characterized by an inner radius (*R*_i_, which corresponds to *R*_mag_) and the width of the flow (*W*_*r*_) at the disk plane. The model assumes a steady-flow prescription for a given accretion rate to determine the density at a given point. The temperature at each point is determined parametrically, scaled to the density assuming a constant heating rate in the flow; the maximum temperature in the flow (*T*_max_) describes each model. To calculate the emission line profile, the model assumes the extended Sobolev approximation and calculates the mean intensity and the level population of a 16-level hydrogen atom, and uses the ray-by-ray method for a given viewing inclination (*i*).

We created a large grid of models varying the accretion rate, *R*_mag_, *W*_*r*_, *T*_max_ and *i*, using the ranges of parameters appropriate for accreting T Tauri stars^49^. With these combinations, we calculated around 72,000 model profiles. We convolved the model profiles with a Gaussian instrumental profile of CHIRON’s resolution and fitted each observed profile inside ±400 km s^−1^ from the line centre. The best fits are determined by calculating the *χ*^2^ for each combination of the model and observed profile.

For each observed profile, we selected 100 best fits and calculated the means of the accretion rate, *R*_mag_, *W*_*r*_, *T*_max_ and *i* (Extended Data Table 3). The accretion rates listed in Extended Data Table 3 are higher on the dates for which there are peaks in the UV emission. The derived *i* are roughly consistent with the measured inclination of the disk, which is 53°^17^. *W*_*r*_ varies between 0.2*R*_star_ and 0.5*R*_star_; *T*_max_ varies between 8,270 K and 9,120 K. We use the derived *R*_mag_ of GM Aur to compare it to simulations in the next section.

In Extended Data Fig. 3, we show the best-fitting model to the Hα profiles. The fit to the line wings is very good. The regions with strong absorption features on the blue side of the line were excluded from the fit; this blueshifted absorption is probably from winds, which are not included in the model. There is no periodic pattern in the Hα line (Extended Data Fig. 3). However, multicomponent high-velocity blueshifted absorption, starting on December 7, occurs along with peaks in the UV emission (Fig. 1) and accretion rate (Extended Data Tables 2, 3). There is a possible second component at higher, blueshifted velocity on December 6, which might imply a deceleration after the launching of a higher-density outflow event that may presage the accretion event on December 7. Models^51^ show that there is a disk-wind component to the Hα profile, which manifests as blueshifted absorption; this component is dominated by the innermost disk, within tens of stellar radii.

### 3D MHD simulations of accretion

Here we show global 3D MHD simulations of a rotating magnetized star accreting from a disk^5,23,25^. In brief, the models assume that the star has a dipole magnetic field with a misalignment angle (*θ*) between the rotation axis (*Ω*) and magnetic axis (*μ*). The rotation axes of the star and disk are aligned. Here we use simulations with the same set up as previous work^23,25^, with parameters chosen to approximate the properties of GM Aur.

We assume that the magnetic field of GM Aur can be approximated with a dipole field. The hot spot of GM Aur is at a high latitude of about 77°, measured from radial velocity variations of He i (5,876 Å)^52^; previous work also found that the hot spot is at high latitudes for dipole–octupole configurations^1^. Also, GM Aur has a long rotation period^21^, and young stars with simpler, more dipole magnetic fields are slower rotators^53^. Simulations^23^ show that the properties of the hot spot depend on *θ*, the corotation radius (*R*_co_) and *R*_mag_. For GM Aur, *θ* = 13°^52^. This is consistent with the range of inclinations of the accretion flow inferred from modelling the Hα profile (all the best-fitting inclinations in Extended Data Table 3 are within about 10° of the system inclination of 53°). Using a stellar rotation period of 6.1 days^21^, *R*_star_ = 2*R*_sun_ and *M*_star_ = 1.36*M*_sun_^47^ (where *R*_sun_ and *M*_sun_ are the radius and mass of the Sun), *R*_co_ = 7.8*R*_star_ (0.07 au). We measure a mean *R*_mag_ of about 3.8*R*_star_, with a range of 3.4*R*_star_ to 4.6*R*_star_ (Extended Data Table 3). The simulations use *θ* = 20°, *R*_mag_ ≈ 4.5*R*_star_ and *R*_co_ = 5.7*R*_star_, which are consistent with the parameters of GM Aur.

The 3D MHD simulations have an approximate accretion rate of about 1.1 × 10^−8^*M*_sun_ yr^−1^, which agrees with measurements from the accretion-shock and accretion-flow modelling (Extended Data Tables 2, 3). The energy distribution in the spots in the 3D MHD simulations varies between about 3.9 × 10^9^ erg s^−1^ cm^−2^ and 1.2 × 10^11^ erg s^−1^ cm^−2^; this is consistent with the accretion-shock models, which use energy fluxes of 1 × 10^10^ erg s^−1^ cm^−2^ to 1 × 10^12^ erg s^−1^ cm^−2^. The densities of the 3D MHD simulated spots in Fig. 4 range between about 5.5 × 10^−13^ g cm^−3^ and 5.97 × 10^−12^ g cm^−3^, which overlaps with the accretion-shock models, where the densities are 2.1 × 10^−13^ g cm^−3^ to 2.1 × 10^−11^ g cm^−3^. The simulated light curves peak at a luminosity of about 1 × 10^31^ erg s^−1^ (Fig. 4), which is roughly consistent with the accretion luminosities of about 0.1*L*_sun_ to 0.2*L*_sun_ (3.8 × 10^32^ erg s^−1^ to 7.7 × 10^32^ erg s^−1^; *L*_sun_ is the luminosity of the Sun) measured from the HST spectra. In Fig. 4 we show the light curve only once accretion onto the star begins in the simulation. To generate the light curve for the densest region of the hot spot, we use *ρ* > 6.5 × 10^−12^ g cm^−3^.

A ratio of *R*_co_/*R*_mag_ = 1.5 sets the boundary between stable and unstable accretion^25^. In the stable regime, matter accretes onto the star in ordered funnel streams, and symmetric, crescent-shaped hot spots are expected^54^. In the unstable regime, matter accretes in chaotic hot spots^25^. The simulations have *R*_co_/*R*_mag_ = 1.3–1.4. The measured *R*_mag_ of GM Aur has an uncertainty of about 1*R*_star_, so *R*_co_/*R*_mag_ for GM Aur could reach 1.4. Therefore, the simulations are consistent with the properties of GM Aur, and both are near the boundary of the stable–unstable regime. Here, processes at the disk and magnetosphere boundary may cause the behaviour seen in the observed light curves. The inner disk rotates more rapidly than the magnetosphere, leading to non-stationary behaviour of matter in the inner disk and corresponding non-stationary behaviour of the hot spot. The hot spots are predominantly crescent-shaped, but may become asymmetric (Fig. 4). Also resulting from the difference in the disk and magnetosphere rotation, the non-stationarity leads to variation of the density distribution in the hot spot that especially influences the densest parts of the spot, which may occasionally disappear. This may explain why the UV emission (associated with the high-density region of the hot spot) disappears in Fig. 1.

## Online content

Any methods, additional references, Nature Research reporting summaries, source data, extended data, supplementary information, acknowledgements, peer review information; details of author contributions and competing interests; and statements of data and code availability are available at 10.1038/s41586-021-03751-5.

## Figures and Tables

**Extended Data Table 1:**
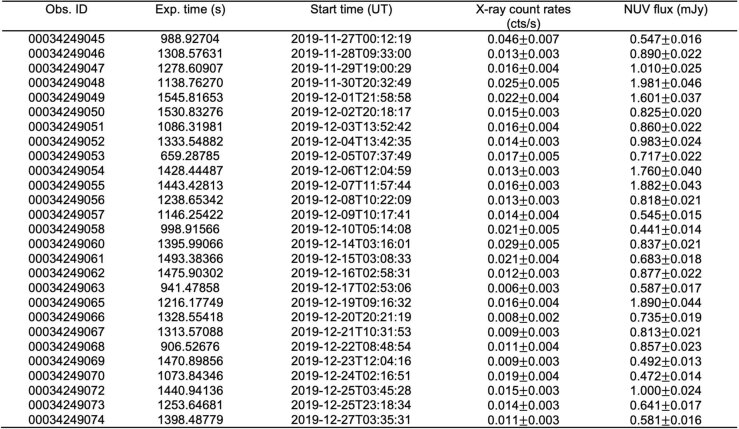
Swift observations and fluxes

**Extended Data Table 2:**
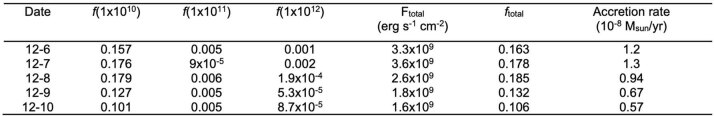
Accretion-shock model parameters

**Extended Data Table 3:**
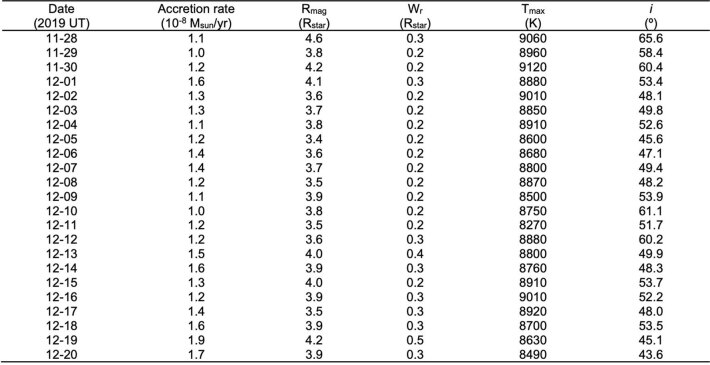
Accretion-flow model parameters

UT, universal time.

## Data Availability

The raw data for the Swift, HST, TESS (https://archive.stsci.edu) and LCOGT (https://lco.global/) data are publicly available from their respective archives. The LDT and CHIRON raw data are available on request from C.C.E. HST and TESS provide reduced data in their archives. Reduced Swift, LCOGT, CHIRON and LDT data are available on request from C.C.E. Data from the accretion-shock and accretion-flow modelling are available on request from C.C.E. Data relevant to the 3D MHD numerical simulations are available on request from M.M.R. (romanova@astro.cornell.edu).

## Code availability

Access to the accretion-shock, accretion-flow and MHD model codes are available on request from N.C. (ncalvet@umich.edu), J.M. (muzerol@stsci.edu) and M.M.R., respectively.
